# The effects of postoperative malrotation alignment on outcomes of Gartland type III/IV paediatric supracondylar humeral fractures treated by close reduction and percutaneous K-wire fixation

**DOI:** 10.1186/s13018-023-04505-x

**Published:** 2024-01-03

**Authors:** Cao Chen, Yafeng Zhang, Hao Chen, Jie Sun, Chen Yao

**Affiliations:** Department of Orthopaedics, Affiliated Hospital of Nantong University, Medical School of Nantong University, 20 Xisi Road, Nantong City, 226001 Jiangsu Province People’s Republic of China

**Keywords:** Supracondylar humeral fracture, Gartland type III/IV, Malrotation, Outcomes

## Abstract

**Purpose:**

In this study, we aimed to investigate the effects of postoperative malrotation alignment on the outcomes of Gartland type III/IV paediatric supracondylar humeral fracture (SCHF) treated by close reduction and percutaneous K-wire fixation.

**Methods:**

Between January 2014 and December 2021, 295 Gartland type III/IV paediatric SCHFs treated by close reduction and percutaneous K-wire fixation were selected for this retrospective study. The demographic, clinical and radiographic parameters of all cases were collected. The lateral rotation percentage (LRP) was measured on X-rays to evaluate postoperative malrotation alignment of the fracture. All cases were categorized into 4 groups according to LRP: LRP ≤ 10% (210, 71.2%), 10% < LRP ≤ 20% (41, 13.9%), 20% < LRP ≤ 30% (26, 8.8%) and LRP > 30% (18, 6.1%). The carrying angle, ranges of multidirectional motions, Mayo Elbow Performance Score (MEPS) and Flynn’s Standard Score (FSS) of the injured elbow were assessed 6 months postoperation and compared among different groups. ROC analysis based on LRP and the excellent/good rate of FSS was performed to determine the acceptable maximum degree of postoperative malrotation alignment.

**Results:**

There was no difference in the demographic characteristics (age, sex, injured side and fracture type), postoperative Baumann angle, carrying angle or range of forearm rotation among the 4 groups (*P* > 0.05). The operation time and time from operation to K-wire removal were longer in the 20% < LRP ≤ 30% and LRP > 30% groups than in the LRP < 10% and 10% < LRP ≤ 20% groups (*P* < 0.001). The shaft condylar angle, range of elbow flexion, MEPS and FSS of the injured elbow 6 months postoperatively were lower in the 20% < LRP ≤ 30% and LRP > 30% groups than in the LRP < 10% and 10% < LRP ≤ 20% groups (*P* < 0.001). ROC analysis based on LRP and the excellent/good rate of FSS showed an area under the curve of 0.959 (95% CI 0.936–0.983), with a cutoff value of 26.5%, sensitivity of 95.3% and specificity of 90.1%.

**Conclusion:**

A certain degree of residual malrotation alignment deformity of the SCHF may reduce the shaft condylar angle and extend the time from operation to removing the K-wire and affect elbow function, especially the range of elbow flexion. The acceptable maximum degree of residual malrotation deformity expressed as the LRP value was 26.5%.

## Background

Supracondylar humeral fracture (SCHF) is the most common type of elbow fracture in children (accounting for 65%) and the second most common paediatric fracture (accounting for 3%) [1, 2]. Most SCHFs are extension fractures (accounting for 97–99%) [3, 4], which are classified into four types by Gartland et al. according to the degree of displacement and stability [5, 6]. Type III/IV SCHFs are characterized by complete displacement and instability, which account for 45% of all cases in Chinese children [3]. Sometimes type III/IV SCHFs may be combined with neurovascular injuries and emergency operations with fracture reduction and fixation are recommended to ease pain and reduce complications [7]. Although the conservative treatment and open reduction have also been reported, close reduction and percutaneous fixation with Kirschner wire (K-wire) is the most common option for type III/IV SCHFs [3, 8, 9].

Due to the features of type III/IV SCHFs and the limited experience of young doctors on duty, achieving satisfactory reduction during emergency operations is challenging. The surgeon can correct and estimate the deformity of angulation, displacement or shortening according to some elbow parameters, such as the Baumann angle and the shaft condylar angle (SCA), on plain X-ray photography during the operation. However, it is relatively difficult to correct the malrotation alignment. It was reported that only 26% of type III SCHFs achieve rotational stability with two lateral K-wires [10]. Sometimes, postoperative malrotation alignment is the main complication of SCHF. Traditionally, the remodelling capacity of the paediatric humerus and the large range of motion of upper limb joints can compensate for malreduction [11]. Actually, the distal humerus provides only 20% of humeral growth [1], which may be finite. However, unacceptable malrotation alignment may still exist, which is ignored by most orthopaedic surgeons.

CT scan is the gold standard of bony deformity evaluation, but it may cause too much radiation to children and is not a routine examination for SCHF in our hospital. However, it is difficult to quantify the rotational alignment on plain X-ray photography. Henderson provided an arithmetic approach to calculate the ratio of rotational deformity [12], but the protocol is too complex. Noora Tuomilehto et al. registered malrotation if the reduction of the medial and lateral columns was asymmetric, which was quite rough [13]. Chen JC et al. determined the malrotation alignment when the anterior humeral line passed the anterior one-third of the capitellum on the lateral X-ray and reported that a certain degree of rotational deformity had little effect on outcomes [14], which was inaccurate and unreliable. Gordon JE et al. first used the lateral rotational percentage (LRP) to reflect the degree of rotational deformity of SCHFs [15]. As an easy and valid parameter, LRP was calculated by dividing the absolute amount of displacement of the proximal humeral metaphysis by the width of the distal humerus at the fracture site on the standard lateral X-ray. Furthermore, Berdis G et al. confirmed that there was a near linear correlation between the degree of malrotation and the LRP [16]. However, the relationship between various degrees of postoperative malrotation alignment and the clinical outcomes and what degree of rotational deformity can be acceptable remain unclear.

In this study, we retrospectively collected all type III/IV SCHFs treated with close reduction and percutaneous K-wire fixation in our institution, categorized them into different groups based on LRP and compared the clinical outcomes of the fractures among these groups. Our results will help to understand the clinical effect of postoperative malrotation alignment and determine the acceptable maximum degree of malrotation by LRP, providing a clinical reference for orthopaedic surgeons.

## Materials and methods

### Patients

We conducted the retrospective study in the Traumatic Orthopaedics of the Affiliated Hospital of Nantong University, which was approved by the Clinical Research Ethics Committee of the Institution and complied with the principles in the declarations of Helsinki. All paediatric patients with SCHF who came to our institution between January 2014 and December 2021 were evaluated for this study after the informed consent was signed by their legal guardians. The inclusion criteria were as follows: fresh Gartland type III/IV SCHF, treated by close reduction and percutaneous K-wire fixation within 48 h after injury, aged 2–12 years old, with complete follow-up data. Notably, type IV SCHF is a complete fracture with multidirectional stability due to incompetent periosteal hinge, which should be diagnosed intraoperatively. All the cases in our study were assessed and classified during the operation according to the literature published by Leitch KK et al. [6]. During the operation, if the surgeon confirmed that the fracture could displace into either flexion or extension easily with slight pressure, it was considered multidirectional unstable and classified as type IV. The exclusion criteria were as follows: Gartland type I/II SCHF, old fracture, open fracture, pathological fracture, neurovascular injuries or other concomitant injuries of the ipsilateral leg, mental illness or other medical conditions before injury, age younger than 2 years or older than 12 years, and incomplete follow-up data. In our hospital, all patients with SCHF routinely underwent outpatient follow-up at 6 weeks, 3 months and 6 months postoperatively. The clinical evaluation of elbow function and radiologic evaluation of the fracture were performed and recorded routinely at each follow-up. In this study, we retrospectively reviewed and analysed the existing data to reach some conclusions.

### Group design according to postoperative malrotation

**Fig. 1:**
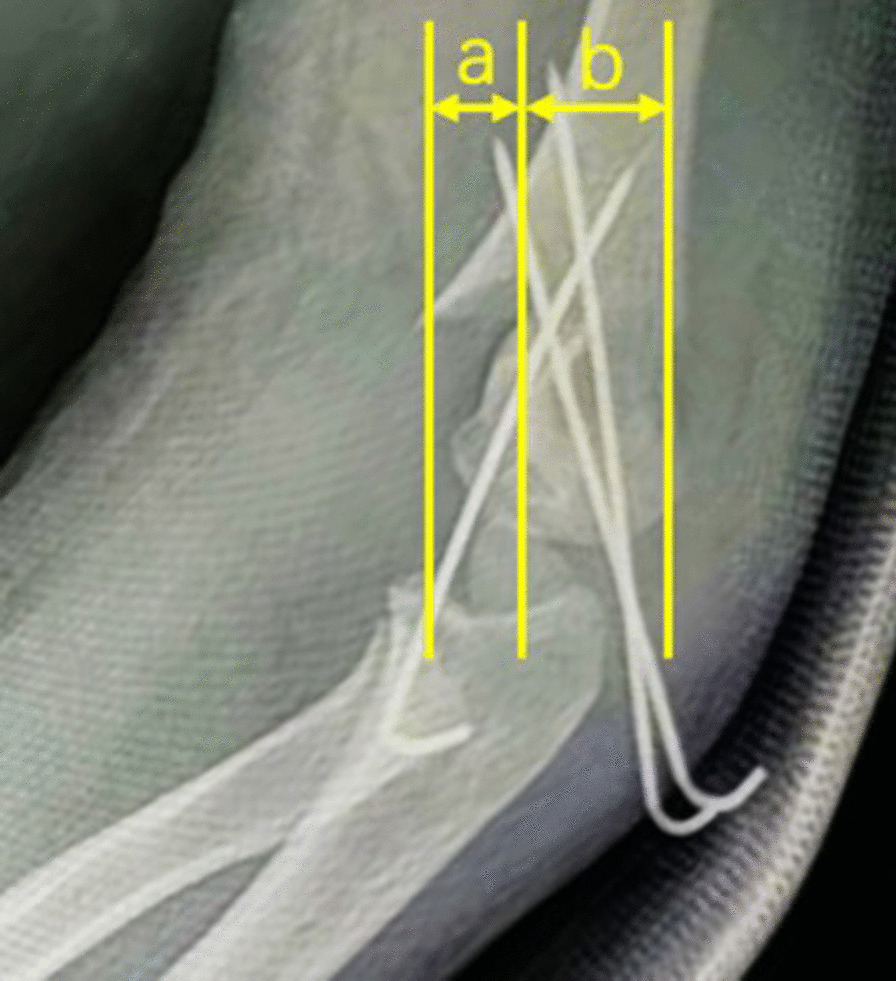
Measurement of LRP. On the lateral X-ray, the forward shift of the proximal humeral cortex **a** was divided by the width of the distal humerus **b** at the fracture site, and the value was multiplied by 100 to be the LRP

**Fig. 2:**
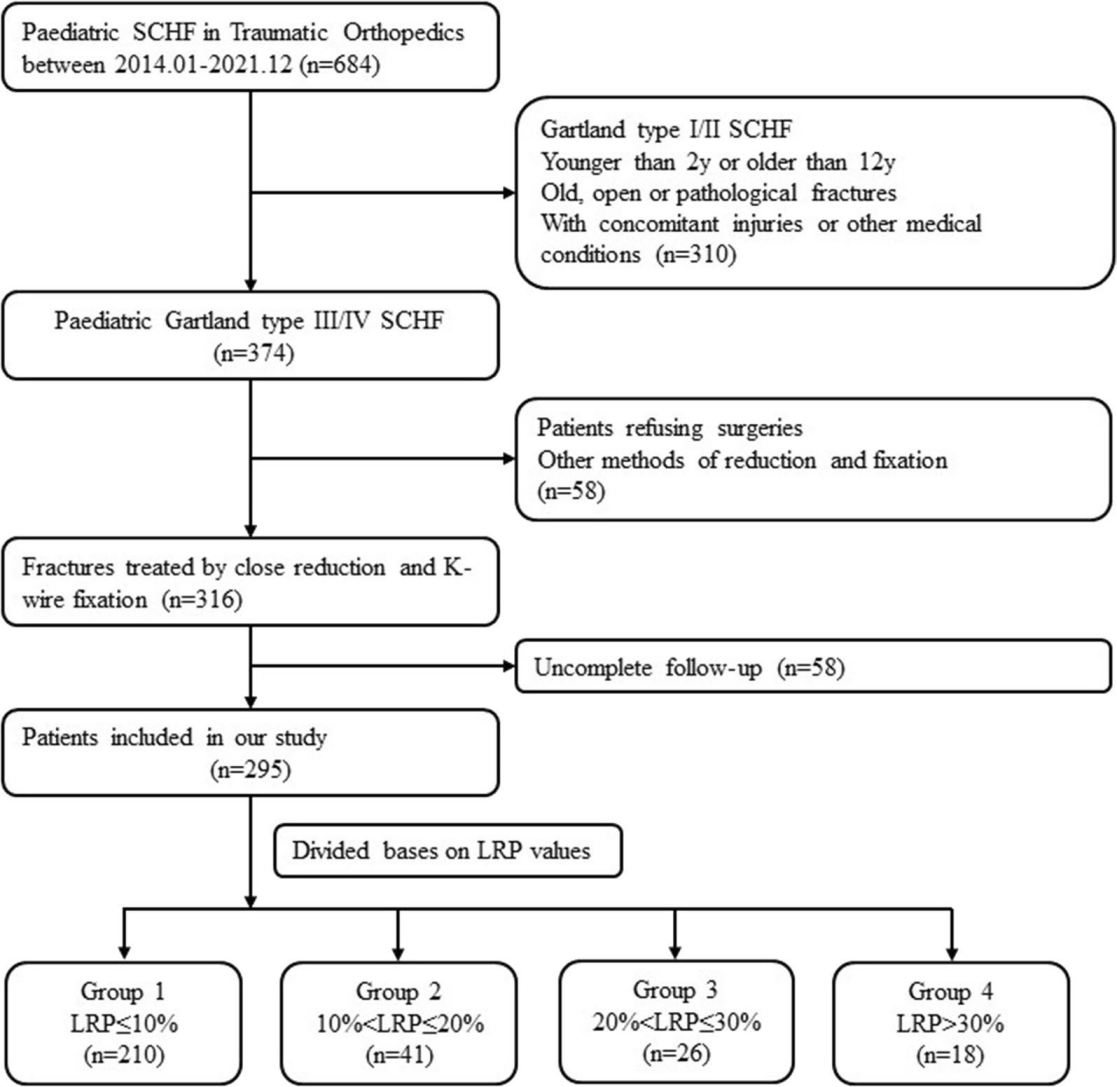
Flowchart of participants throughout the study

Referring to the method reported by Gordon JE et al. [15], we evaluated the degree of malrotation by measuring the LRP on the standard lateral X-ray postoperatively. Obtaining a good true lateral view is challenging, especially for those cases with malrotation alignment postoperatively. Normally, our imaging technicians try to place the distal part of the fracture site on the lateral view with overlapping of the medial and lateral humeral condyles as much as possible to obtain a standard lateral X-ray of the elbow. Those cases with an unacceptable lateral X-ray were excluded by researchers during the retrospective study. The LRP was calculated by dividing the absolute amount of displacement of the proximal humeral metaphysis by the width of the distal humerus at the fracture site as shown in Fig. 1. To minimize interobserver bias, each X-ray was measured by two surgeons independently, and an average value was obtained. Then, the assessment was checked by the third surgeon to ensure the reliability of the measurement. Then, all the cases were categorized into four groups according to the degree of malrotation deformity (LRP): LRP ≤ 10%, 10% < LRP ≤ 20%, 20% < LRP ≤ 30%, and LRP > 30%. The flowchart of the participants in this study is shown in Fig. 2.

### Data collection and follow-up assessments

**Fig. 3:**
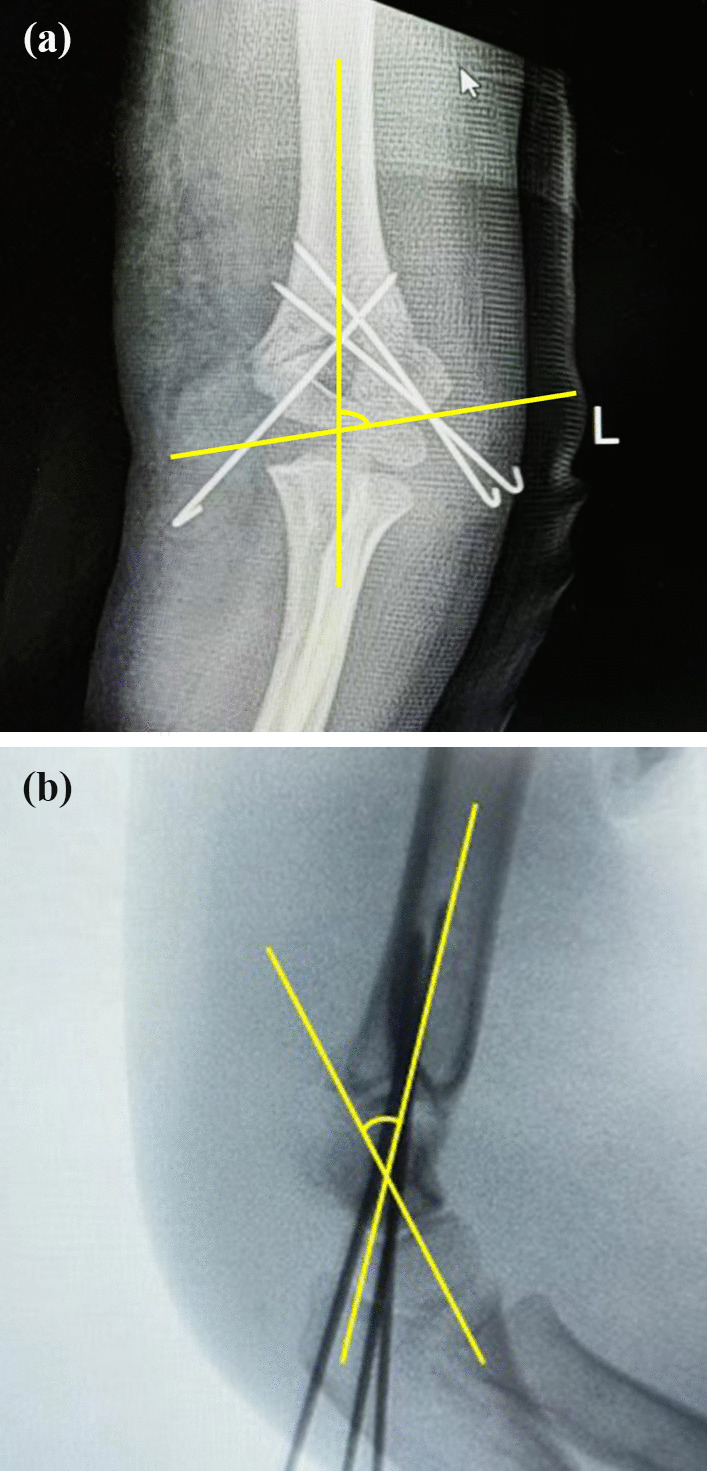
Measurement of the Baumann angle (**a**) and SCA (**b**) on the frontal and lateral X-rays, respectively

**Fig. 4:**
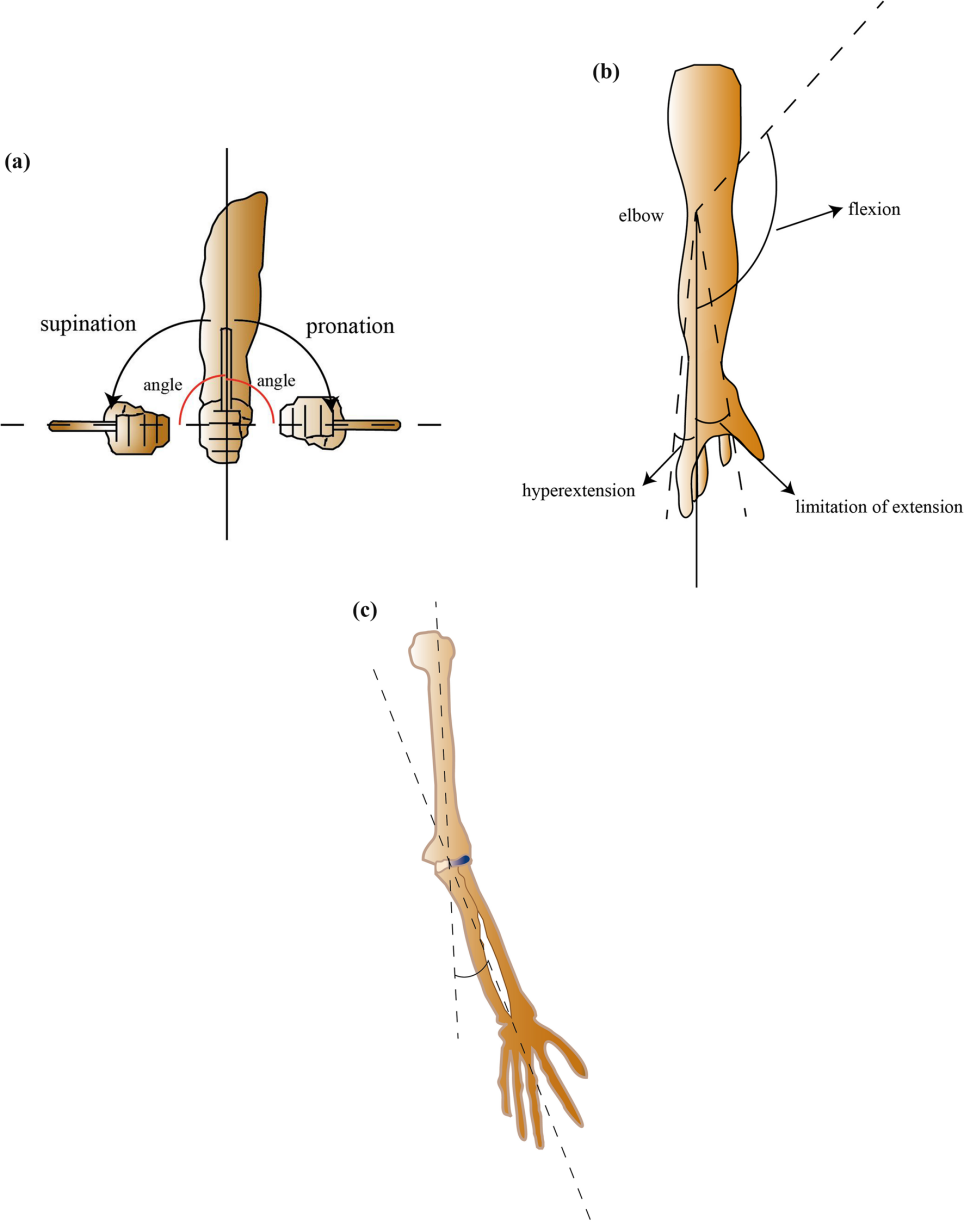
Measurement of ranges of multidirectional motions and the carrying angle of the injured elbow. **a** Forearm pronation and supination; **b** elbow flexion, limitation of elbow extension (negative value) and elbow hyperextension (positive value); **c** the carrying angle

The demographic and clinical characteristics, radiographic data and functional outcomes were collected and compared among these four groups. Baseline characteristics included age, sex, injured side and Gartland type of fracture. Perioperative data included hospital stay, hospitalization cost, operation time and time to remove the K-wire. The Baumann angle and SCA of the elbow were measured on plain films 1 day and 6 months after the operation (Fig. 3). The carrying angle and multidirectional range of motion (ROM) of the elbow, including flexion, extension, pronation and supination of the forearm, were measured 6 months postoperation (Fig. 4). The patients' elbow functions were evaluated according to the Mayo Elbow Performance Score (MEPS) and Flynn's Standard Score (FSS) 6 months postoperatively.

### Statistical analysis

Statistical analysis was performed with SPSS 15.0 software (SPSS Inc., Chicago, IL, USA). The Kolmogorov‒Smirnov test was used to evaluate the normal distribution. Continuous variables are expressed as the mean ± standard deviation (normal distribution) or median, 25th and 75th percentiles (nonnormal distribution). The categorical variables were expressed as numbers with percentages. One-way ANOVA was applied to compare the continuous variables among different groups. The test of equal variance for continuous variables was previously performed. Modified *P* values calculated by SPSS were adapted when equal variance was not assumed in some continuous variables. The Chi-square test and Fisher’s test were applied according to the sample size to compare the categorical variables among different groups. ROC analysis was performed to determine the acceptable maximum degree of postoperative malrotation based on LRP and the excellent/good rate of FSS. A *P* value less than 0.05 was considered statistically significant.

## Results

Finally, a total of 295 children with Gartland type III/IV SCHF who accepted close reduction and percutaneous K-wire fixation were included in this study. They were distributed as follows: 210 (71.2%) in the LRP ≤ 10% group, 41 (13.9%) in the 10% < LRP ≤ 20% group, 26 (8.8%) in the 20% < LRP ≤ 30% group and 18 (6.1%) in the LRP > 30% group. The baseline demographic and clinical characteristics of the cases are summarized in Table 1. There were no significant differences in age, sex, injured side or proportion of Gartland type among the four groups. Significantly, the duration of surgery and the time to remove the Kirschner wire after surgery were longer in the 20% < LRP ≤ 30% group and the LRP > 30% group than in the LRP < 10% group and the 10% < LRP ≤ 20% group (*P* < 0.001).

**Table 1:** Baseline demographic and clinical characteristics of the cases in different groups

Subjects	Groups				*P*
	Group 1 (*n* = 210) LRP ≤ 10%	Group 2 (*n* = 41) 10% < LRP ≤ 20%	Group 3 (*n* = 26) 20% < LRP ≤ 30%	Group 4 (*n* = 18) LRP > 30%	
Age, year (mean ± SD)	6.83 ± 2.93	5.91 ± 3.17	7.35 ± 2.85	6.19 ± 2.47	0.121
Sex, *n*%					
Boys	115 (54.76%)	20 (48.78%)	15 (57.69%)	11 (61.11%)	0.670
Girls	95 (45.23%)	21 (51.22%)	11 (42.3%)	7 (38.88%)	
Side of injury, *n*%					
Left	106 (50.47%)	24 (58.54%)	15 (57.69%)	8 (44.44%)	0.671
Right	104 (49.52%)	17 (41.46%)	11 (42.30%)	10 (55.56%)	
Gartland type, *n*%					
III	86 (41.24%)	26 (63.42%)	11 (42.30%)	9 (50%)	0.072
IV	124 (59.04%)	15 (36.59%)	15 (57.69%)	9 (50%)	
Operation time, minute (mean ± SD)	46.12 ± 10.66	43.59 ± 9.68	59.27 ± 10.71	56.75 ± 9.46	< 0.001
Time to remove K-wire, day (mean ± SD)	30.03 ± 5.30	33.68 ± 6.75	50.97 ± 6.54	48.29 ± 6.01	< 0.001

There were no significant differences in the Baumann angle measured on anterior–posterior X-ray photography 1 day or 6 months after the operation among the four groups. However, the SCA measured on lateral X-ray photography 1 day or 6 months after the operation in the 20% < LRP ≤ 30% group and in the LRP > 30% group was significantly smaller than that in the LRP < 10% group and in the 10% < LRP ≤ 20% group (*P* < 0.001); details of the results are summarized in Table 2.

**Table 2:** Baumann angle, SCA and carrying angle of the injured elbow 1 day or 6 months postoperatively in different groups

Subjects	Groups				*P*
	Group 1 (*n* = 210) LRP ≤ 10%	Group 2 (*n* = 41) 10% < LRP ≤ 20%	Group 3 (*n* = 26) 20% < LRP ≤ 30%	Group 4 (*n* = 18) LRP > 30%	
Baumann angle 1 day postoperatively, degree (mean ± SD)	75.12 ± 7.52	75.17 ± 8.492	76.52 ± 9.42	76.54 ± 6.91	0.664
Baumann angle 6 months postoperatively, degree (mean ± SD)	73.62 ± 8.98	74.1 ± 6.67	72.73 ± 7.91	75.61 ± 7.70	0.586
SCA 1 day postoperatively, degree (mean ± SD)	30.53 ± 3.85	30.76 ± 5.25	26.94 ± 10.53	24.07 ± 9.21	< 0.001
SCA 6 months postoperatively, degree (mean ± SD)	31.31 ± 6.43	30.59 ± 4.85	27.97 ± 10.12	27.11 ± 8.23	< 0.001
Carrying angle 6 months postoperatively, degree (mean ± SD)	12.57 ± 4.54	14.01 ± 6.97	11.89 ± 5.16	13.81 ± 4.77	0.474

No significant difference was detected in the carrying angle, ranges of forearm rotation or extension of the injured elbow 6 months postoperatively. However, the ranges of elbow flexion 6 months postoperatively in the 20% < LRP ≤ 30% group and the LRP > 30% group were significantly smaller than those in the LRP < 10% group and the 10% < LRP ≤ 20% group; details of the results are expressed in Table 3.

**Table 3:** The ranges of multidirectional motions of the elbow and forearm 6 months postoperatively in different groups

Subjects	Groups				*P*
	Group 1 (*n* = 210) LRP ≤ 10%	Group 2 (*n* = 41) 10% < LRP ≤ 20%	Group 3 (*n* = 26) 20% < LRP ≤ 30%	Group 4 (*n* = 18) LRP > 30%	
Ranges of forearm pronation, degree (mean ± SD)	84.64 ± 2.35	85.44 ± 2.28	85.85 ± 2.09	85.04 ± 2.14	0.084
Ranges of forearm supination, degree (mean ± SD)	85.72 ± 3.85	84.46 ± 2.75	85.27 ± 2.74	86.74 ± 3.73	0.066
Ranges of elbow flexion, degree (mean ± SD)	137.17 ± 3.88	135.61 ± 2.93	130.58 ± 2.60	126.44 ± 3.39	< 0.001
Ranges of elbow extension, degree (mean ± SD)	2.99 ± 1.32	2.61 ± 1.16	2.80 ± 1.18	2.40 ± 1.79	< 0.621

The MEPS in the LRP < 10% group and the 10% < LRP ≤ 20% group was 99.77 ± 1.86 and 98.29 ± 5.32, respectively, with excellent rates and good rates of 100%, which are significantly higher than those in the 20% < LRP ≤ 30% group and the LRP > 30% group. The details of the results are shown in Table 4.

**Table 4:** The MEPS of the injured elbow 6 months postoperatively in different groups

Subjects	Groups				*P*	*P*_i–j_
	Group 1 (*n* = 210) LRP ≤ 10%	Group 2 (*n* = 41) 10% < LRP ≤ 20%	Group 3 (*n* = 26) 20% < LRP ≤ 30%	Group 4 (*n* = 18) LRP > 30%		*P*_1–2_ = 0.033
MEPS (mean ± SD)	99.77 ± 1.86	98.29 ± 5.32	94.39 ± 11.16	84.82 ± 8.76	< 0.001	P_1–3_ < 0.001
Levels of MEPS (*n*%)						
Excellent	206 (98.10%)	38 (92.68%)	20 (76.92%)	4 (22.22%)	< 0.001	P_1–4_ < 0.001
Good	4 (1.90%)	3 (7.32%)	2 (7.69%)	11 (61.11%)		P_2–3_ = 0.049
Fair	0 (0%)	0 (0%)	4 (15.38%)	8 (16.67%)		P_2–4_ < 0.001
Poor	0 (0%)	0 (0%)	0 (0%)	0 (0%)		P_3–4_ < 0.001

As shown in Table 5, the FSS 6 months postoperatively in the 20% < LRP ≤ 30% group and the LRP > 30% group was significantly smaller than those in the LRP < 10% group and the 10% < LRP ≤ 20% group (*P* < 0.001), mainly due to the limitation of elbow flexion and extension.

**Table 5:** The FSS of the injured elbow 6 months postoperatively in different groups

Subjects	Groups				*P*	*P*_i–j_
	Group 1 (*n* = 210) LRP ≤ 10%	Group 2 (*n* = 41) 10% < LRP ≤ 20%	Group 3 (*n* = 26) 20% < LRP ≤ 30%	Group 4 (*n* = 18) LRP > 30%		
Levels of FSS (*n*%)						
Excellent	193 (91.9%)	11 (26.83%)	2 (7.69%)	1 (5.56%)	< 0.001	*P*_1–2_ < 0.001 *P*_1–3_ < 0.001 *P*_1–4_ < 0.001 *P*_2–3_ = 0.043 *P*_2–4_ < 0.001 *P*_3–4_ = 0.0375
Good	17 (8.10%)	21 (51.2%)	15 (57.69%)	8 (44.44%)		
Fair	0 (0%)	2 (21.95%)	5 (19.23%)	7 (38.89%)		
Poor	0 (0%)	0 (0%)	4 (15.38%)	2 (11.11%)		

**Fig. 5:**
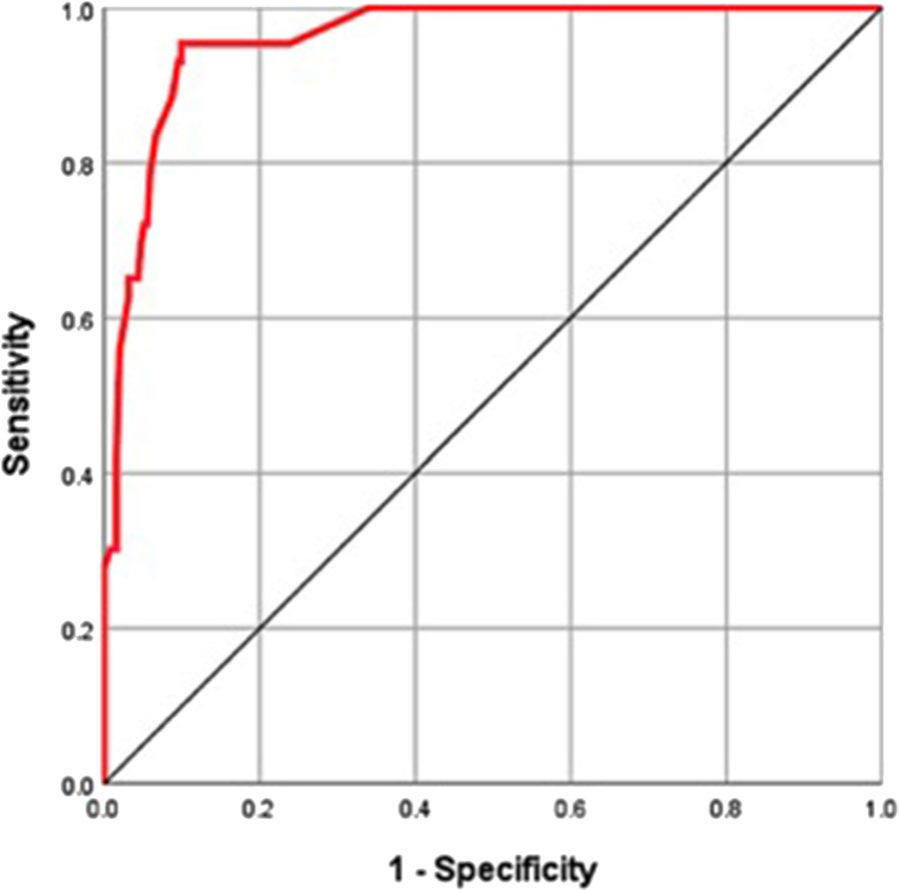
The ROC curve analysis based on LRP and the excellent/good rate of FSS

ROC analysis according to LRP and the excellent/good rate of FSS showed an area under the ROC curve AUC of 0.959 (95% CI 0.936–0.983) with a cutoff value of 26.5%, sensitivity of 95.3% and specificity of 90.1% (Fig. 5), indicating that the acceptable maximum degree of postoperative residual rotational displacement expressed as the LRP value should not exceed 26.5%.

## Discussion

Postoperative rotational deformity is an important feature of Gartland type III/IV SCHF. In our study, the cases with a postoperative LRP of more than 20% accounted for approximately 15% of all cases, and this observation needs to be given more attention. The purpose of this study was to investigate the effect of postoperative malrotation on the functional outcomes of Gartland type III and IV paediatric SCHFs treated with close reduction and percutaneous K-wire fixation. We categorized subjects into four groups according to malrotation deformity degrees based on the value of LRP and found that patients in the 20% < LRP ≤ 30% and LRP > 30% groups had longer operation times and longer times to remove the K-wire, limited ranges of elbow flexion and extension, lower MEPS and lower FSS 6 months postoperatively. Additionally, we conducted ROC analysis according to LRP and FSS, revealing that the LRP should not exceed 26.5%, providing the first report of the acceptable maximum degree of postoperative malrotation deformity.

In this study, we found that the operation time in the 20% < LRP ≤ 30% and LRP > 30% groups was longer than that in the LRP < 10% and 10% < LRP ≤ 20% groups. This suggested the challenge of rotational reduction and reminded us that making more attempts during the operation may be useless. If we cannot find more effective methods of rotational reduction to correct the malrotation deformity, we must become clear about the influence of residual malrotation deformity on fracture outcomes and judge whether it is worth pursuing perfect rotational reduction. On the other hand, the cases in the 20% < LRP ≤ 30% and LRP > 30% groups exhibited longer times to remove the K-wire, which suggested that a larger malrotation deformity may influence fracture healing. The smaller contact area of the fracture site caused by malrotation deformity may be one of the possible reasons. The malrotation deformity also made the fracture line more obvious on the lateral X-rays, and it may take a longer time for the orthopaedists to conform to the disappearance of the fracture line during postoperative follow-up, delaying the removal of the K-wire. Luckily, in all the cases, fracture healing was achieved within 60 days, without delayed union or nonunion, consistent with a previous study. The growth potential of paediatric bones eliminated our worries about fracture healing with residual malrotation deformity.

The Baumann angle and carrying angle are important elbow parameters on the frontal view and are related to cubitus varus [17]. Cubitus varus is considered the most common long-term complication of SCHF, which usually needs to be corrected by osteotomy for cosmetic reasons [18]. A previous study reported that malrotation deformity may be one of the causes related to cubitus varus [19]. In contrast, we found that there were no significant differences in the postoperative Baumann angle or carrying angle among the four groups in our study. Compared with other parameters, surgeons may preferentially pay more attention to correcting the abnormal Baumann angle to avoid postoperative cubitus varus, which is relatively easy to achieve through close reduction according to intraoperative fluoroscopy. However, the SCA in the 20% < LRP ≤ 30% and LRP > 30% groups were smaller than that in the LRP < 10% and 10% < LRP ≤ 20% groups. The SCA is the distal corner of the anatomical axis of the humerus and anteroposterior axis of the lateral condyle, which is an important reference for sagittal plane correction in SCHF [20]. When we place the humeral condyles on the standard lateral view, the distal part of the humeral proximal to the fracture site is wider, and the anatomical axis of the distal humeral is forward due to the residual malrotation deformity, which may cause a reduction in SCA. Actually, SCA is very difficult to evaluate or correct accurately during the operation, particularly when malrotation deformity exists.

Multidirectional ROMs of the elbow are vital functional outcomes of SCHF. We found that there was no difference in forearm rotation or extension of the injured elbow among the different Groups 6 months postoperatively. However, the range of elbow flexion 6 months postoperatively in the 20% < LRP ≤ 30% group and the LRP > 30% group was significantly smaller, which is the main factor for the lower MEPS and FSS in these two groups. The relationship between ROMs of the elbow and fracture reduction is still controversial. Simanovsky N et al. thought that sagittal malreduction of the SCHF may limit elbow flexion [21]. Paradis G et al. reported that the late function of the elbow depended primarily on the reduction quality [22]. In contrast, Rachel S Silverstein et al. reported that there was no association between malrotation deformity and postoperative ROMs [23].

When malrotation deformity exists, we hypothesize that the anterior impingement of the fragment rotating forward may limit elbow flexion. The patients with a larger LRP had a longer time for K-wire removal and suffered a longer time of elbow fixation of casting, which delayed the early functional rehabilitation of the elbow. This may be another factor of the limited elbow ROMs. Regardless, the loss of elbow flexion or extension is minimal and may be compensated by other joints.

Age is an important factor related to the remodelling potential of bony deformities in paediatric fractures. The healing ability of supracondylar humeral fractures in children of different ages varies greatly. Gamble JG et al. reported that children under 5 years of age can remodel even a 100% displacement of the fracture, whereas those older than 8 years have limited remodelling capacity [24]. However, our study did not examine the potential correlation with respect to the age of patients. Due to the limited number of cases, we cannot further categorize patients into subgroups by different ages. If so, then there could be even fewer cases with a higher LRP in each subgroup, possibly affecting the reliability of the statistical analysis. Hopefully, in the future, when we collect adequate cases, we will try to analyse the different effects of malrotation alignment on elbow function in different age groups.

In addition to LRP, elbow function postoperatively may be affected by several other factors, such as age, complications, duration of immobilization, and loss of reduction. These factors may also affect each other. We grouped all cases according to LRP and compared age, sex, injury side, fracture type, Baumann angle, carrying angle and some other available information among different groups, aiming to find some confounding factors. However, this is a retrospective study of existing case data over the past 8 years. We are afraid that some other confounding factors could have been neglected at the follow-ups. Moreover, the number of cases with poor elbow function was too small. Therefore, we did not group the samples according to elbow function scores and perform multivariate regression analysis to adjust the effect of LRP considering all possible confounding factors. This limitation will be avoided in the future when we accumulate adequate cases and more detailed case data.

Several other limitations of our study exist and need to be mentioned. Among all 295 patients included in this study, the sample size of Groups 3 and 4 with a higher LRP, totalling 44 patients, was relatively small. During the operation, the surgeons always made their best effort to achieve a satisfactory or acceptable reduction of fractures, except in some cases which were especially difficult. We considered that this is why the number of samples with a higher LRP is relatively small. CT scanning is the gold standard for bony deformity evaluation, but it may cause too much radiation in children. Here, we measure the LRP on lateral X-rays of the elbow to quantify the malrotation deformity indirectly, which, as an approach, is relatively easy and safe but inaccurate compared with CT scanning. Taking a standard lateral X-ray is limited by the expertise of radiographic technicians and the cooperation of children. Malrotation deformities include pronation and supination which are hard to distinguish on plain films. In addition to the parameters mentioned in our study, malrotation deformity may cause other kinds of deformities, such as humeral torsion reported by Anna K. Hell et al. [25]. Additionally, we only conducted follow-up for 6 months after the operation, which is relatively short considering the ongoing skeletal growth in a paediatric population. The functional outcomes of paediatric SCHF may be different after 6 months due to continued bone growth. Most patients in our hospital underwent K-wire removal approximately 6 weeks after the operation and achieved full functional recovery at the 6-month follow-up. After that, most of them will not return to our hospital, so additional follow-up data cannot be obtained. Longer follow-up needs to be conducted in the future to achieve more comprehensive reporting.

## Conclusion

In summary, our study showed a certain degree of residual malrotation deformity of the SCHF after the operation which may reduce the SCA on lateral X-rays and extend the time to remove the K-wire. The function of the elbow, especially the range of flexion, may be affected by malrotation deformity 6 months postoperatively. The acceptable maximum degree of residual malrotation deformity expressed as the LRP value was 26.5%.

## Data Availability

Please contact authors for data requests.

## Abbreviations

FSS: Flynn’s Standard Score
K-wire: Kirschner wire
LRP: Lateral rotation percentage
MEPS: Mayo Elbow Performance Score
ROC: Receiver-operating characteristic
ROM: Range of motion
SCA: Shaft condylar angle
SCHF: Supracondylar humeral fracture
